# A body mass index over 22 kg/m^2^ at college age is a risk factor for future diabetes in Japanese men

**DOI:** 10.1371/journal.pone.0211067

**Published:** 2019-01-24

**Authors:** Yuki Someya, Yoshifumi Tamura, Yoshimitsu Kohmura, Kazuhiro Aoki, Sachio Kawai, Hiroyuki Daida, Hisashi Naito

**Affiliations:** 1 Sportology Center, Juntendo University Graduate School of Medicine, Tokyo, Japan; 2 Department of Metabolism & Endocrinology, Juntendo University Graduate School of Medicine, Tokyo, Japan; 3 Juntendo University Graduate School of Health and Sports Science, Chiba, Japan; 4 Faculty of Health and Sports Science, Juntendo University, Chiba, Japan; 5 Department of Cardiology, Juntendo University Graduate School of Medicine, Tokyo, Japan; Universidade de Sao Paulo, BRAZIL

## Abstract

**Background:**

There is a high incidence of type 2 diabetes in Asian adults, even those with a normal body mass index (BMI) (<25.0 kg/m^2^). For example, it has been shown that a slightly increased BMI (>23 kg/m^2^) at middle age is a risk factor for type 2 diabetes in Asians. In this historical cohort study, we investigated whether a slightly increased BMI at college age was also a risk factor for future diabetes in Japanese men.

**Methods:**

Six hundred and sixty-one male alumni who graduated from a physical education school between 1971 and 1991 and who responded to follow-up investigation between 2007 and 2017 were included in this study. Participants were categorized into four categories: college BMI of <21.0 kg/m^2^, 21.0–22.0 kg/m^2^, 22.0–23.0 kg/m^2^, and ≥23.0 kg/m^2^, and the incidence and risk ratio of diabetes were compared between groups.

**Results:**

The median follow-up period was 32 years (interquartile range, IQR: 27–36), which included 20,983 person-years of observation. Subjects were 22 (22–22) years old at college graduation, and 55 (50–59) years old at the final follow-up investigation. During the study period, 56 men developed diabetes; the prevalence rates for the lowest to highest BMI categories were 4.4%, 7.6%, 10.5%, and 11.3%, respectively, and their adjusted hazard ratios were 1.00 (reference), 1.77 (95% CI: 0.68–4.30), 2.42 (1.00–5.84), and 2.53 (1.06–6.07), respectively (p = 0.03 for trend).

**Conclusion:**

Our data suggest that a BMI over 22.0 kg/m^2^ at college age is a risk factor for diabetes later in life in Japanese men.

## Introduction

According to the World Health Organization (WHO), diabetes is associated with 1.6 million deaths per year and is recognized as one of the top 10 causes of death worldwide [1]. Overweight and obesity, defined by body mass indexes (BMIs) of more than 25 and 30 kg/m^2^, respectively, have been reported as risk factors for not only type 2 diabetes, but also for hypertension, dyslipidemia, cardiovascular disease (CVD), and mortality [2–6]. The prevalence of type 2 diabetes is rapidly increasing in Asian countries, and patients with diabetes in Asia are characterized by a lower BMI than those in Western countries; for example, the mean BMI of Asian patients with type 2 diabetes was reported to be approximately 23 kg/m^2^ [7]. Further, metabolic disorders are also more common in Asians than in BMI-matched Caucasians [8, 9]. In fact, a slightly increased BMI (>23 kg/m^2^) in middle age (around 50 years old) is recognized as a risk factor for the development of type 2 diabetes in Koreans and Japanese [10, 11]. Accordingly, the American Diabetes Association recommends a BMI cutoff value of 23.0 kg/m^2^ for screening of type 2 diabetes in Asian Americans [12].

Some reports have demonstrated that BMI at a young age predicted future metabolic disorders. For example, overweight, obesity, and high weight-for-height at ~20 years of age have all been found to be risk factors for the development of type 2 diabetes at middle age [13, 14]. Very recently, it has been shown that being overweight at 7 and 13 years of age and early adulthood (17 to 26 years of age) was positively associated with the risk of developing type 2 diabetes at age 30 and older [15]. Although we and others showed that slightly increased BMI (approximately 1 kg/m^2^) at 20 years of age increased the risk of future hypertension in Japanese men even if the future BMI was within the normal range [16, 17], it is unknown whether slightly elevated BMI at a young age also increases the risk of future diabetes.

To investigate this question, we performed a historical cohort study and analyzed the database of a Juntendo University Alumni Study [16, 18, 19]. We used the standardized physical fitness examination data of the alumni recorded when they were college students, and investigated the association between BMI at a young age and development of diabetes later in life.

## Methods

### Study participants

The data of this study were derived from the Juntendo University Alumni Study. Subjects were male alumni of the Physical Education School of Juntendo University who graduated from 1956 to 1991. In this cohort, we previously demonstrated that physical fitness and physical characteristics at the college age level were associated with future diseases such as diabetes and hypertension [16, 18, 19]. The number of male alumni who participated at least once in follow-up investigations between 2007 and 2017 was 1,348 (Flowchart of subjects was appeared in our previous reports [16, 18, 19]). However, alumni who graduated before the institution of the standardized physical examination in 1971 (n = 661) or who had missing data (n = 23) were excluded from this analysis. Of the remaining alumni, three males did not appropriately complete the questionnaire and were therefore excluded from the study. In addition, female alumni were excluded because there were too few subjects before 1991. Thus, we included 661 male alumni in the final analysis.

The study protocol was approved by the Juntendo University Ethics Committee in 2007 (Nos. 19–131) and 2016 (Nos. 28–30). All data were anonymized before analysis to ensure participant privacy. Along with the questionnaire, we sent a letter of informed consent approving the collection and use of the athletic test data for research purposes. We obtained consent from all subjects involved in the study. This study was carried out in accordance with the principles outlined in the Declaration of Helsinki.

### Assessment of BMI at college age

Juntendo University students have undergone standardized examinations of physical characteristics and fitness annually since 1971, and all data have been stored. We used data on participants’ height and body weight to calculate their average BMI during their college years.

### Determination of prevalence of diabetes

The prevalence of diabetes was determined based on self-administered questionnaires completed between 2007 and 2017. In the survey, alumni were asked whether they were diagnosed with diabetes after graduation from college, and if so, the age at which they were diagnosed by a physician [16, 18, 19]. In this study, the follow-up period began at college graduation and ended at the diagnosis of diabetes or the final follow-up examination.

### Statistical analysis

We categorized participants into four categories based on BMI at college age: <21.0 kg/m^2^ (n = 155), 21.0–22.0 kg/m^2^ (n = 172), 22.0–23.0 kg/m^2^ (n = 170), and ≥23.0 kg/m^2^ (n = 160). The distribution of participants in categories was similar when participants were divided by quartiles. We defined the lowest BMI group (BMI <21.0 kg/m^2^) as the reference category. The prevalence rates of diabetes among BMI categories were compared by the χ^2^ test. Hazard ratios and 95% confidence intervals (95% CIs) relating BMI to the incidence risk of diabetes were constructed using Cox proportional hazards analysis. The unadjusted and multivariable-adjusted relative risks were computed. The adjusted data included age at graduation (continuous variable, 1-year-old increments), year of graduation (continuous variable, 1-year increments), and smoking (category, yes or no). All data were obtained at college age. The year of graduation was used as a surrogate variable for economic status. All statistical analyses were conducted using SPSS version 23.0 for Windows software (SPSS Inc., Chicago, IL).　

## Results

### Characteristics of study participants

The characteristics of participants in each BMI category are shown in Table 1. The median age at college graduation was 22 (interquartile range, IQR: 22–22) years old. The median BMI at college age was within the normal range (<25.0 kg/m^2^) in each category, and was only 24.0 (IQR: 23.4–25.2) kg/m^2^ even in the highest BMI group. Thus, only 36 participants (5.4%) were overweight (BMI ≥25 to <30 kg/m^2^) and five (0.8%) were obese (BMI ≥30 kg/m^2^). Almost all of the participants had participated in college sports. The percentage of smokers was comparable between groups.

**Table 1:** Baseline characteristics according to BMI categories in former male college athletes.

	<21 kg/m^2^ (n = 158)	21–22 kg/m^2^ (n = 172)	22–23 kg/m^2^ (n = 172)	≥23 kg/m^2^ (n = 159)	All (n = 661)
Age, years	22 (22–22)	22 (22–22)	22 (22–22)	22 (22–22)	22 (22–22)
Year of graduation	1982 (1978–1985)	1982 (1978–1986)	1982 (1979–1986)	1982 (1978–1986)	1982 (1978–1986)
Body mass index, kg/m^2^	20.4 (19.8–20.7)	21.5 (21.2–21.8)	22.4 (22.2–22.7)	24.0 (23.4–25.2)	22.0 (21.1–23.0)
Smoker, n (%)	77 (48.7)	84 (48.7)	91 (52.9)	73 (45.9)	325 (49.2)
College sports participation, n (%)	157 (99.4)	167 (97.1)	169 (98.3)	156 (98.1)	649 (98.2)

Data are at college age and are median (IQR: interquartile range), number (%), or column percentage.

### Association between BMI and incidence of diabetes

The follow-up period of this study was 32 years (IQR: 27–36 years), which included 20,983 person-years of observation. The median age and BMI at the time of the final follow-up questionnaire were 55 years (IQR: 50–59 years) and 23.9 kg/m^2^ (IQR: 22.2–25.6 kg/m^2^), respectively. During the follow-up period, 56 men developed diabetes, the unadjusted cumulative incidence of diabetes was 8.5%, and the median age at diagnosis was 51 years (IQR: 45.3–57.0 years).

**Fig 1:**
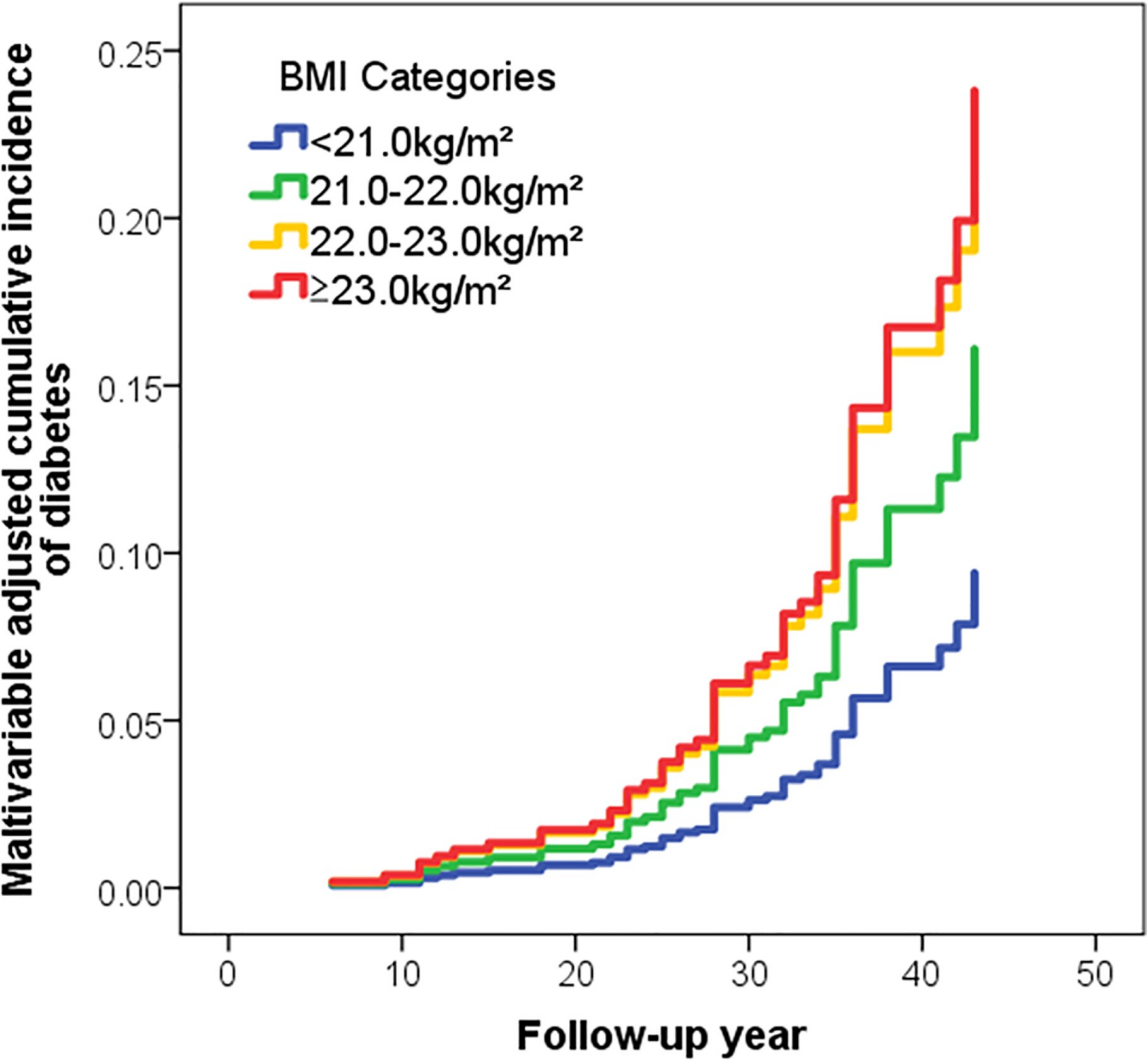
Multivariable-adjusted cumulative incidence rate curves for diabetes during follow-up according to BMI categories.

We evaluated the incidence rate and hazard ratio of diabetes in each category to determine the relationship between BMI at college age and the development of diabetes later in life. Both the incidence of diabetes per 10,000 person-years and the unadjusted hazard ratio were positively associated with BMI at college age (Table 2). This association persisted after adjustment for age, year of graduation, and smoking (Table 2). In addition, the risk of diabetes in two BMI categories, namely 22.0–23.0 kg/m^2^ and over 23.0 kg/m^2^, was significantly higher than in the under-21.0 kg/m^2^ category. The multivariable-adjusted cumulative incidence rate of diabetes was also positively associated with BMI throughout the follow-up period, and the trends in the 22.0–23.0 kg/m^2^ and ≥23 kg/m^2^ BMI categories were similar (Fig 1). These results suggest that a BMI over 22.0 kg/m^2^ at college age might be a risk factor for future diabetes in Japanese men.

**Table 2:** Hazard ratio for diabetes according to BMI.

	BMI categories				
	<21 kg/m^2^	21–22 kg/m^2^	22–23 kg/m^2^	≥23 kg/m^2^	p for trend
Number	158	172	172	159	
Follow-up duration (years)	32 (29–36)	32 (27–36)	32 (27–36)	31 (27–36)	
Person-years of follow-up	5122	5487	5381	4993	
Diagnosed with diabetes (n, %)	7 (4.4)	13 (7.6)	18 (10.5)	18 (11.3)	
Incidence per 10,000 person-years	13.7	23.7	33.5	36.1	
Age at diagnosis (years)	54 (49–57)	56 (49–61)	53 (45–57)	47 (39–53)	
Unadjusted hazard ratio (95% CI)	1.00	1.72 (0.69–4.32)	2.39 (0.99–5.77)	2.62 (1.09–6.27)	0.02
Multivariable adjusted hazard ratio* (95% CI)	1.00	1.77 (0.68–4.30)	2.42 (1.00–5.84)	2.53 (1.06–6.07)	0.03
Age (years)	0.93 (0.51–1.70)				0.82
Year of graduation	0.95 (0.88–1.02)				0.13
Smoker (yes/no)	0.73 (0.43–1.25)				0.26

*Cox proportional hazards models adjusted for baseline characteristics (age, year of graduation, and smoking).

## Discussion

In this study, we investigated the relationship between BMI at college age and the future development of diabetes in Japanese male alumni of a physical education school. Hazard ratios for diabetes in the BMI categories of 22–23 kg/m^2^ and over 23 kg/m^2^, adjusted for potential confounding factors, were 2.4 and 2.5 times higher, respectively, than that for the BMI category of under 21.0 kg/m^2^. These data suggest that a slightly increased BMI at college age, even when within the normal range, may be associated with the future development of diabetes in Japanese men.

A recent report showed that being overweight at 7 years old, 13 years old, or early adulthood (17 to 26 years old) increased the risk of future type 2 diabetes [15]. An additional risk factor was a high weight-for-height at ~20 years of age [14]. However, we found that even a very low BMI (22–23 kg/m^2^) at college age conferred an increased risk of future diabetes. In term of hypertension, we and others previously showed that a slightly increased BMI (more than 23.0 kg/m^2^ or 21.0 kg/m^2^) at around age 20 increased the risk of future hypertension [16, 17]. These data suggest that BMI at a young age can predict future metabolic disease in Japanese, even when it is within the normal range.

It has been suggested that the BMI cut-off point for an increased risk of type 2 diabetes in middle-age Asian Americans should be 23.0 kg/m^2^ or lower [12]. Korean subjects with a BMI ≥23 kg/m^2^ at middle age (around 50 years old) were found to have approximately double the risk of type 2 diabetes compared with subjects with a BMI from 18.5 to 22.9 kg/m^2^ [10]. We demonstrated that a relatively low BMI (only 22–23 kg/m^2^) at college age increased the risk of diabetes later in life; however, a normal BMI at a young age might rise to over 23 kg/m^2^ later in life, thus becoming a risk factor for diabetes. It has been demonstrated that BMI at all ages is positively correlated with BMI later in life [20, 21]. In fact, our preliminary analysis revealed that BMI at a young age was correlated with BMI at follow-up examination (age around 55 years), and subjects with a BMI over 21.0 kg/m^2^ at a young age had a BMI over 23.0 kg/m^2^ at middle age.

The exact mechanism whereby relatively low BMI at college age increases the risk of diabetes is unclear, but recent data demonstrated that in non-obese Asians, this risk is elevated not only by impaired insulin secretion, but also by insulin resistance. Insulin secretion in type 2 diabetes is lower in Asians than in Caucasians, and therefore slightly increased insulin resistance may result in glucose intolerance in Asians [22–24]. Asians were shown to have a higher body fat content and more visceral adipose tissue than Caucasians and African Americans of similar BMI [25, 26]. In addition, muscle insulin resistance was observed even in non-obese, non-diabetic subjects with one or more cardiometabolic risk factors, and was associated with visceral adiposity [27].

The present study has several limitations. First, the study enrolled only male alumni of one university department, and almost all were former college athletes. Although subjects lived all over Japan at the time of the study, they were not representative of all Japanese men. Second, we could not precisely diagnose diabetes and its types (e.g. type 1 or type 2). In Japan, the prevalence of type 2 diabetes is assumed to be 100 times higher than type 1 diabetes, thus we supposed that most of diabetes participants declared in the current study might be type 2 diabetes. Third, we analyzed male alumni who responded to follow-up questionnaires, and therefore self-selection bias may have occurred. For example, the participants who answered the follow-up questionnaires may have been those who were the healthiest. Nonetheless, the prevalence of diabetes in the current study was similar to the prevalence of type 2 diabetes in the general population [28, 29]. In addition, waist circumference is considered as more sensitive marker for central adiposity and insulin resistance; however, we did not measure it in the current study. Thus, if we use waist circumference instead of BMI, we may more sensitively predict the onset of diabetes. Next, recall bias was also possible because participants were asked to remember their medical history pertaining to diabetes. However, our previous studies used the same method [16, 18, 19] and established the validity of the self-report questionnaires by confirming the data [14]. Finally, this study did not account for changing BMI and lifestyle habits during the follow-up period. When examining the relationship between BMI at a young age and risk of future diabetes, we preliminarily adjusted for current diet, exercise, and abdominal circumference measurement which are importance factor of diabetes, but neither of these factors influenced the results.

## Conclusion

In conclusion, our data suggest that a BMI over 22.0 kg/m^2^ at college age is a significant risk factor for future diabetes in Japanese men.
